# SUANPAN: scalable photonic linear vector machine

**DOI:** 10.1038/s41377-025-02059-7

**Published:** 2026-01-01

**Authors:** Ziyue Yang, Chen Li, Yuqia Ran, Yongzhuo Li, Xue Feng, Kaiyu Cui, Fang Liu, Hao Sun, Wei Zhang, Yu Ye, Fei Qiao, Jiaxing Wang, Cun-Zheng Ning, Connie J. Chang-Hasnain, Yidong Huang

**Affiliations:** 1 Department of Electronic Engineering, Tsinghua University, 100084 Beijing, China; 2 State Key Laboratory for Mesoscopic Physics and Frontiers Science Center for Nano-Optoelectronics, School of Physics, Peking University, 100871 Beijing, China; 3 Berxel Photonics Company Ltd, 518071 Shenzhen, China; 4 College of Integrated Circuits and Optoelectronic Chips, Shenzhen Technology University, 518118 Shenzhen, China

**Keywords:** Optoelectronic devices and components, Photonic devices

## Abstract

Photonics is promising to handle extensive vector multiplications in artificial intelligence (AI) techniques due to natural bosonic parallelism and high-speed information transmission. However, the dimensionality of current photonic linear operation is limited and tough to improve due to the complex beam interaction for implementing optical matrix operation and digital-analog conversions. Here, we propose a programmable and reconfigurable photonic linear vector machine with extreme scalability formed by a series of emitter-detector pairs as the independent basic computing units. The elemental values of two high-dimensional vectors are prepared on emitter-detector pairs by bit encoding and analog detecting method without requiring large-scale analog-to-digital converter or digital-to-analog converter arrays. Since there is no interaction among light beams inside, extreme scalability could be achieved by simply multiplicating the independent emitter-detector pair. The proposed architecture is inspired by the traditional Chinese Suanpan or abacus, and thus is denoted as photonic SUANPAN. Experimentally, the computing fidelities for vector inner products could achieve >98% in our implementation with an 8 × 8 vertical cavity surface emission laser (VCSEL) array and an 8 × 8 MoTe_2_ two-dimensional material photodetector array. Furthermore, such implementation is applied on two typical AI tasks as 1024-dimensional optimization problem is successfully solved and competitive classification accuracy of 88% is achieved for handwritten digit dataset. We believe that the photonic SUANPAN could serve as a fundamental linear vector machine and enhance various future AI applications.

## Abstract (web summary)

SUANPAN: A programmable and reconfigurable photonic linear vector machine with extreme scalability formed by a series of emitter-detector pairs as the independent basic computing units and implemented through time-space encoding.

## Graphical abstract

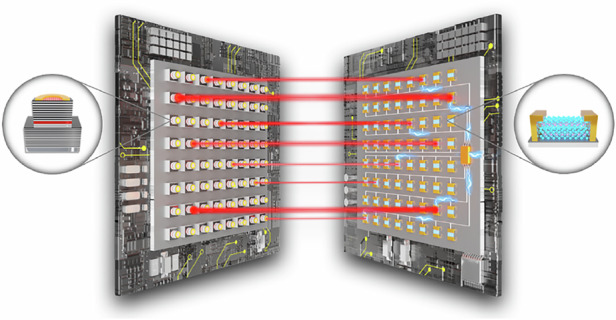

## Introduction

Artificial intelligence (AI) is currently an active topic in both scientific research and commercial applications as well as daily life^1,2^. The linear operations of high-dimensional vectors are fundamental and dominant in both the artificial neural networks^3–5^ (ANN) and optimization problem solvers, *such as* the Ising machine^6–8^. As the complexity of problems increases, the dimensionality of the processed vector grows rapidly, resulting in a huge computational burden. It is known that vector operations can be readily accelerated by photons due to the natural parallelism of bosons^9^. In the past decades, various photonic computing architectures have been demonstrated to perform vector matrix multiplication in the optical domain, i.e., Stanford structure^10–12^, Reck scheme^13–16^, deep diffraction architecture^17–20^, micro-ring resonator (MRR) array^21–26^, etc. All these architectures perform vector matrix multiplications based on the interaction between light beams, which refers to coherent or incoherent superposition between different light beams through beam splitting, beam combining, diffracting, scattering, etc. However, as the optical matrix transformation is adopted, the basic units in the computing architecture, i.e., liquid crystal cells, beam splitters, meta-atoms, etc., would be tightly interconnected or highly coupled with each other due to the interaction. Thus, high-dimensional optical vector-matrix operations cannot be achieved by simply multiplicating these basic units, which significantly limits the scalability of the architecture. Here, instead of optical matrix operations, we propose the SUANPAN architecture for the optical inner product of two vectors. Just like the transistors in an integrated circuit, the independent basic computing unit in our scheme contains only one emitter-detector pair and could be scaled up to form a photonic computing chip. The elemental values of two vectors are encoded on the output intensity of the light-emitters and the photoresponsivity of the photodetectors (PDs), respectively. Thus, the photocurrent of the PD would be proportional to the multiplication of the light intensity and photoresponsivity, and the final result of the inner product can be obtained by the summation of all the photocurrents. Since there is no interaction among the propagating light beams of all emitter-detector pairs and only the output currents of all PDs are connected, our scheme is scalable by increasing the number of emitter-detector pairs with no additional loss or error, as well as flexibly reconfigurable and programmable for different computational tasks.

As a proof of principle, the SUANPAN architecture is implemented by utilizing an 8 × 8 vertical cavity surface emission laser (VCSEL) array and an 8 × 8 MoTe_2_ two-dimensional (2D) material PD array. In the experiment, the calculation fidelity of the random vector inner product can be as high as >98% for various bit precisions (2-bit, 4-bit, and 8-bit), and >95% for various vector dimensionalities (@4-bit precision). Furthermore, such implementation has been successfully reconfigured to perform two typical AI tasks, the Ising machine and the ANN. A randomly generated 1024-dimensional Ising problem is successfully solved, which is the highest dimensionality of optical Ising machine with heuristic algorithm. Meanwhile, a competitive classification accuracy of 88% is achieved for ANN on the MNIST handwritten digit dataset. We believe that our proposed photonic SUANPAN is capable to serve as a fundamental linear vector machine and is potential to enhance the computing power for future various AI applications.

## SUANPAN architecture

**Fig. 1:**
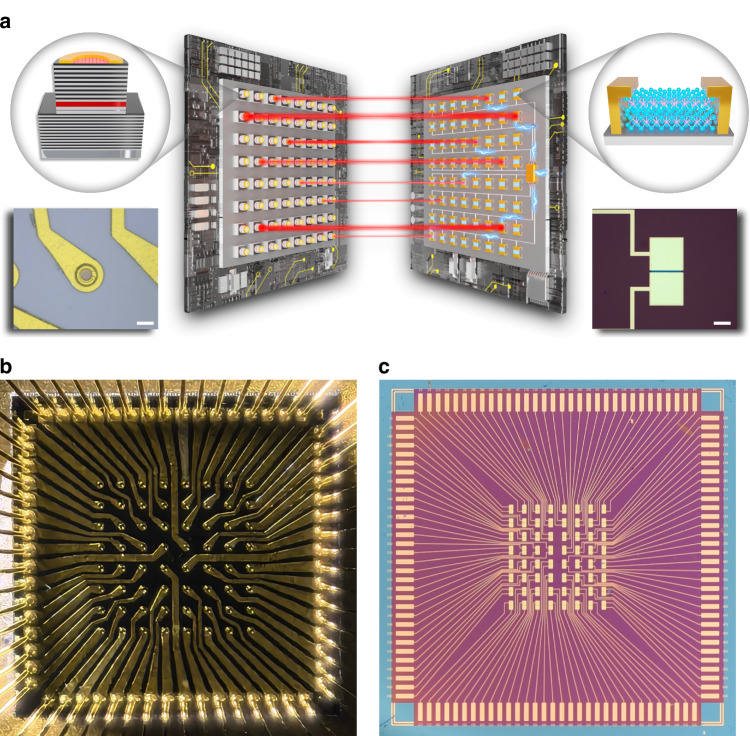
**Architecture of the SUANPAN.** **a** The schematic diagram of the SUANPAN architecture, consisting of a light-emitter array, a PD array, and some necessary electronic hardware. Left insets show the schematic and microscope photograph of a single VCSEL. Scale bar is 20 μm. The right insets show the schematic and microscope photograph of a single MoTe_2_ PD. Scale bar is 100 μm. **b** The optical image of the VCSEL array. **c** The optical image of the MoTe_2_ PD array

The proposed SUANPAN architecture consists of a light-emitter array and a PD array, as well as some necessary electronic hardware, as schematically shown in Fig.1a. To perform a vector inner product, both multiply and accumulate operations are required. Firstly, for multiply operation, each PD is well aligned with a corresponding light-emitter to form an emitter-detector pair, therefore, the photocurrent of the PD would be proportional to the multiplication of the light intensity and photoresponsivity due to the linear optical response^27^. Then, for add operation, all of the PDs are connected so that the output current would be the sum of all PDs due to Kirchhoff’s law. In this way, the multiply-accumulate operation is naturally performed through the emission and detection. Then the key issue is how to encode the vectors on the emitter-detector pairs. A natural way is to directly encode on light intensity and photoresponsivity. However, it would be required that each PD and each emitter should be equipped with a high-precision and high-speed digital-to-analog converter (DAC), which would introduce large power and area overhead^28^ as well as significant latency. Here, we have proposed the Bit Encoding and Analog Detecting paradigm to avoid DAC, thus, one emitter-detector pair is denoted as BEAD.

**Fig. 2:**
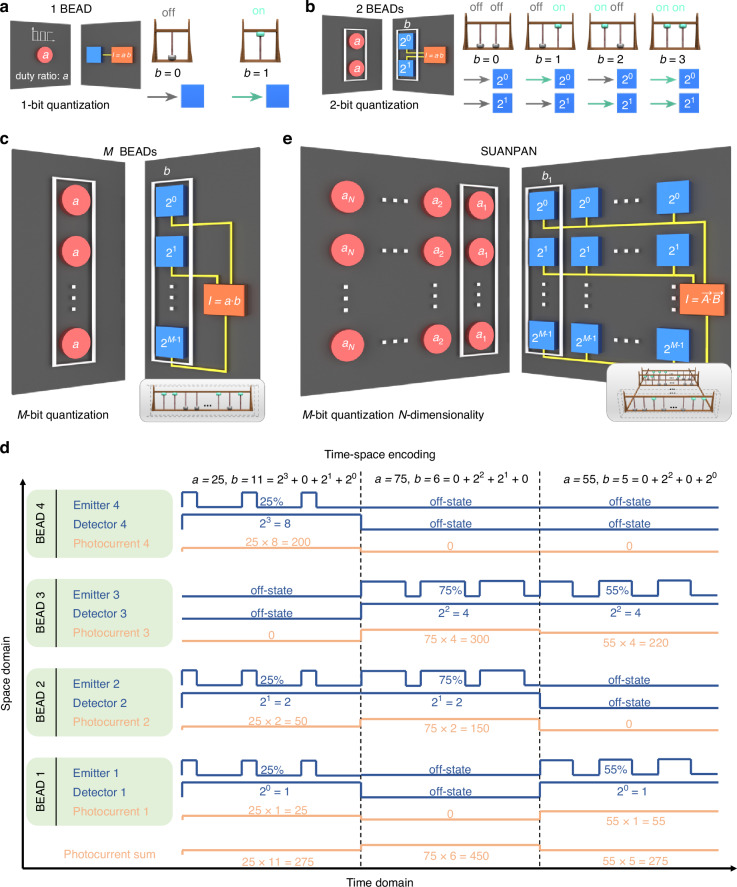
Operation principle of the SUANPAN. **a** The mechanism of performing *a* × *b*, where *b* is 1-bit quantization with 1 BEAD. Right inset: the on-off state of 1 BEAD in the SUANPAN comparing with 1 bead in Chinese Suanpan. **b** The mechanism of performing *a* × *b*, where *b* is 2-bit quantization with 2 BEADs. Right inset: the on-off states of 2 BEADs in the SUANPAN comparing with 2 beads in the Chinese Suanpan. **c** The mechanism of performing *a* × *b*, where *b* is *M*-bit quantization with *M* BEADs. The white box represents a set of *M* BEADs. Inset: *M* beads in Chinese Suanpan as a comparison. **d** An example for the time-space encoding, where *a* = 0, 1, …, 100 by controlling the duty ratio as 0–100%, and *b* is 4-bit quantization by employing 4 BEADs. Since all these 4 detectors are connected, the total photocurrent is proportional to *a* × *b*. **e** The mechanism of performing vector inner product *A·B* with *N* sets of *M* BEADs. Inset: *N* sets of Chinese Suanpan as a comparison

For deep insight, one BEAD is first considered. As shown in Fig. 2a, the multiplier *a* is encoded on the intensity of the light-emitter by controlling the duty ratio of driving current, which is done by a digital counter according to the clock cycles without DAC (the details of encoding shown in Supplementary Note 1 and Fig. S1). The bit precision depends on the time-slot numbers within the period. The multiplier *b* is encoded on the on-off state of the BEAD, by turning on (green arrow) or off (gray arrow) the light-emitter, respectively. Hence, there are two states to encode *b*, *b* = 0 or *b* = 1, known as 1-bit quantization, and the photocurrent would be proportional to *a* × *b*, for *b* = 0, 1. For more bit quantization of *b*, more BEADs are employed to form a set. Considering 2-bit quantization, two BEADs should be employed in one set to obtain four combinations of on-off states. Thus, the two bits in the binary representation of *b* would be corresponding to these two BEADs as shown in Fig. 2b. For example, if *b* = 2, the binary representation would be *b* = 10, which means the first BEAD is at off-state and the second one is at on-state. This operation is quite similar to the Chinese traditional Suanpan, which represents numbers according to the position of beads and carry out mathematic operations by moving the beads up and down. Thus, our scheme is named as photonic SUANPAN. Moreover, different bits represent different weights in binary representation, which can be achieved by setting the photoresponsivity of two PDs as 2^0^–2^1^. To properly manipulate the photoresponsivity, 2D material photoconductive detectors are fabricated. By combining the photocurrents of two PDs, the output result is *a* × *b* as shown in Fig. 2b. In this way, *M*-bit quantization of *b* can be achieved with a set of *M* BEADs as shown in Fig. 2c, so that the SUANPAN can encode the range of *b* from 0 to 2^*M*^−1 and achieve the multiplication of *a* and *b*.

Since *a* is digitally encoded to the duty ratio of light emission and *b* is digitally encoded to the on-off states of the BEADs in one set, while the output result is the total analog photocurrent from all the PDs, the emitter-detector pair is actually operated as Bit Encoding and Analog Detecting. Also, the numbers of *a* and *b* are encoded in time and space domain respectively, therefore, the SUANPAN architecture can perform the multiplication of any desired bit precision theoretically. Figure 2d shows an example of the time-space encoding, where *a* is between 0 and 100 by controlling the duty ratio as 0–100%, and *b* is 4-bit quantization by employing a set of 4 BEADs.

It should be mentioned that the negative numbers can also be handled by applying reversed bias voltage of the PD. Considering both positive and negative numbers, 2 × *M* BEADs are required for each set as shown in Supplementary Note 2 and Fig. S2. Furthermore, the complex vector inner product can also be handled as shown in Supplementary Note 3 and Fig. S3. Thus, the SUANPAN architecture could achieve both reconfigurable and programmable ability, since the number of BEADs in each set can be reconfigured according to the bit precision, while the exact encoding of each BEAD can be flexibly programmed according to the elements within the vectors. At the output, the photocurrents of all PDs are connected, so that only one ADC is required to transform the total photocurrent into a digital signal. Also, since only 1-bit information would be encoded on a single BEAD, the properly settled but fixed bias voltage would be applied. Thus, there is no requirement for DAC in the SUANPAN. Last but not least, since there is no interaction among the propagating light beams of all BEADs, the SUANPAN architecture is scalable by increasing the number of independent BEADs with no additional loss or error. On one hand, the number of utilized BEADs can be increased by integrating more light-emitters and PDs on one chip. On the other hand, distributed computing can also be achieved by simply connecting multiple chips together to scale up the computing power more. Therefore, the SUANPAN is a programmable, reconfigurable, and scalable architecture, which can serve as a general vector inner product accelerator for the existing electronic computing system.

## Results

To implement the prototype of the SUANPAN, a pair of VCSEL and MoTe_2_ PD is employed to form the BEAD. The schematic diagram and microscope photographs of a single VCSEL and MoTe_2_ PD are shown in the insets of Fig. 1a. As a light-emitter, VCSEL can readily achieve high-speed modulation as well as a large-scale array. Recently, researchers have already demonstrated a neural network based on VCSEL^29^. While, in such architecture, each VCSEL requires injection to achieve a stable phase lock. In comparison, for the SUANPAN architecture, all VCSELs are independent. Thus, the phase locking, as well as other additional operations are not required. For PD, 2D material is utilized for three reasons: (1) The photoresponsivity of 2D material PD can be flexibly controlled by the bias voltage. (2) The high carrier mobility in 2D material^30^ can support high-speed detection, which is an important issue for high-speed computing. (3) 2D material can be heterogeneously integrated with other material platform^31^, therefore, 2D material PD is potentially integrated with a light-emitter in the future. Specifically, according to the wavelength of VCSEL (850 nm), MoTe_2_ is utilized as the PD material. Thus, we have fabricated both the VCSEL and MoTe_2_ PD array chip with 8 × 8 components as shown in Fig. 1b and Fig. 1c, respectively. The fabrication process, experimental setup, and performance of VCSEL and PD are shown in Figs. S4–S12 and discussed in Methods. Actually, the BEAD can be achieved with various emitter and detector combinations. Both laser and light emitter diode (LED) could serve as the emitter, while different 2D materials, i.e., graphene^32^, MoS_2_^33,34^, WSe_2_^35–37^, etc. could be applied for PD according to the proper operation wavelength.

**Fig. 3:**
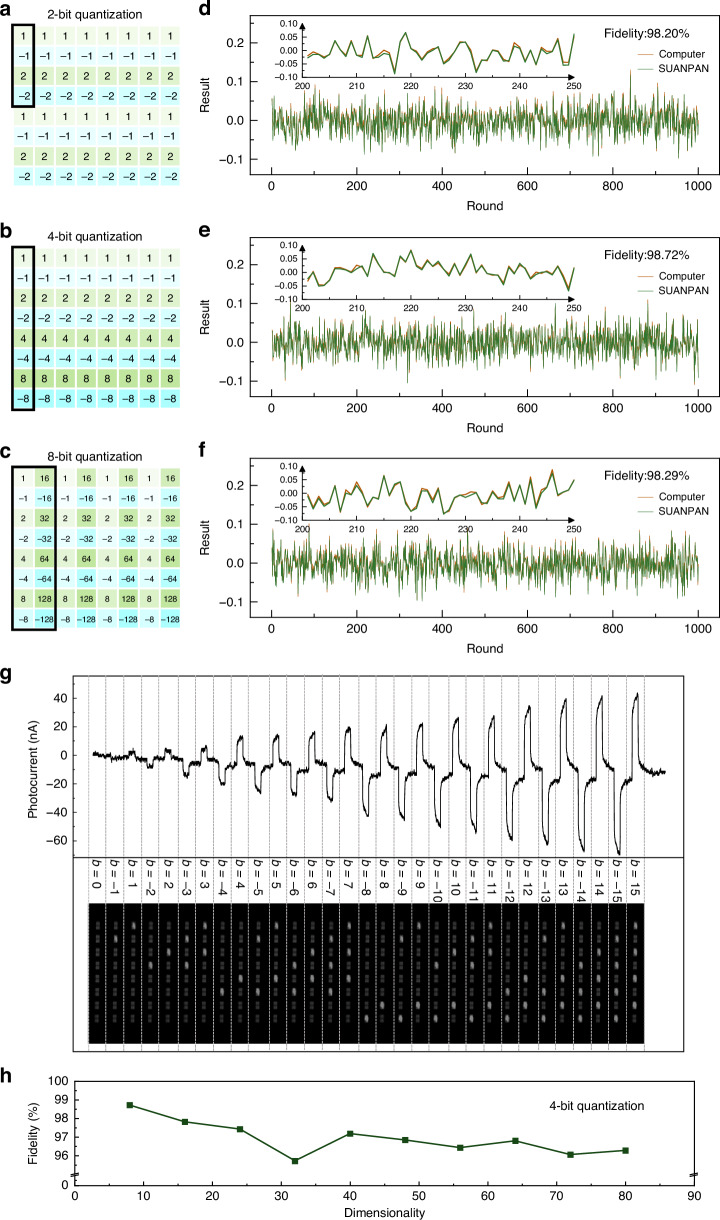
**Random vector inner product.** **a–c** The configuration of the SUANPAN for 2-bit, 4-bit and 8-bit quantization, respectively. **d–f** The normalized results calculated by the SUANPAN and computer for 2-bit, 4-bit and 8-bit quantization, respectively. Insets: the enlarged view of round 201–250. **g** The experimental results of *a* × *b*, where *a* remains unchanged and *b* takes each value in 4-bit quantization. **h** The experimental fidelity for 4-bit quantization with various dimensionalities

To verify the functionality of the SUANPAN, random vector inner products are firstly performed with bit precision of 2-bit, 4-bit, and 8-bit. For a 2-bit precision signed vector, 4 BEADs are required in each set so that a 16-dimensional vector inner product can be done at one time. Similarly, for 4-bit and 8-bit quantization, the corresponding dimensionality would be 8 and 4, respectively. To achieve higher-dimensional vector inner product, time-division multiplexing can also be employed. For each bit precision, the configuration of the SUANPAN would be properly settled and the corresponding bias voltage of each PD is shown in Fig. 3a–c, respectively. Also, 1000 rounds of signed vector inner products are randomly generated and performed by the SUANPAN. To evaluate the accuracy, the normalized results of 1000 rounds for each bit precision calculated by the SUANPAN and computer are shown in Fig. 3d–f, respectively. The achieved fidelities are all higher than 98% (details provided in Supplementary Note 5), which indicates that the SUANPAN can perform accurate calculation.

**Fig. 4:**
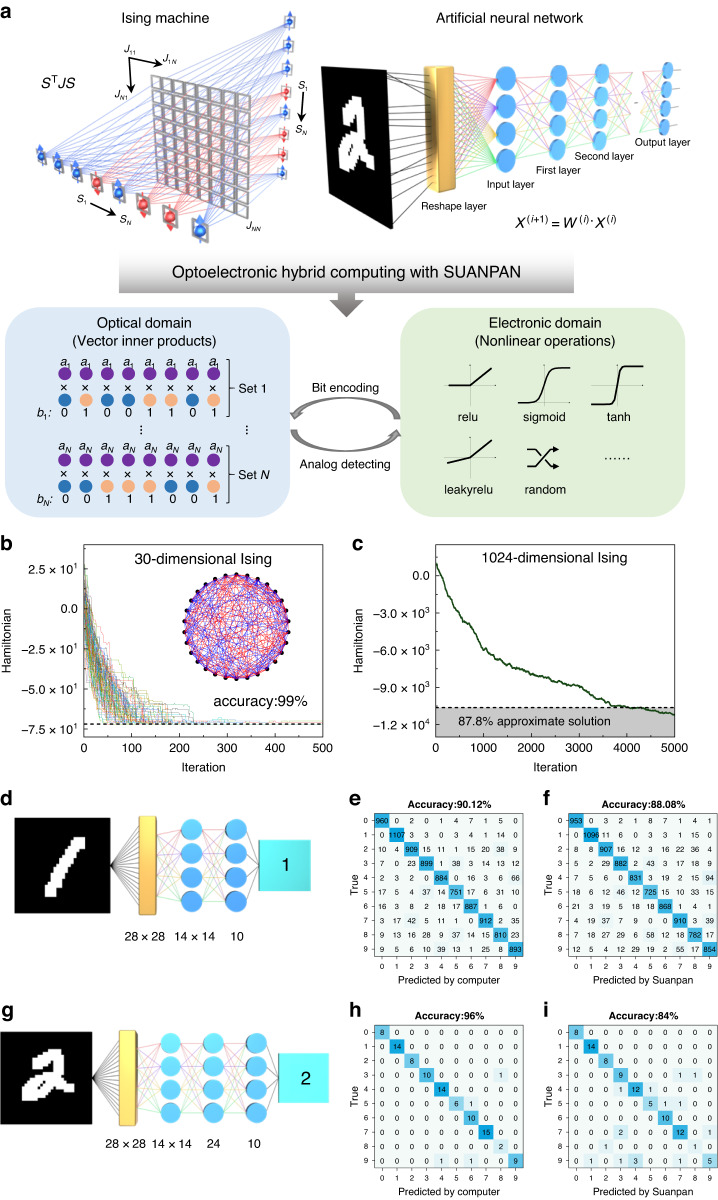
**AI applications.** **a** Various AI applications can be performed on the SUANPAN through optoelectronic hybrid computing. **b** 100 experimental annealing curves of a random 30-dimensional Ising problem. Dashed line: ground state. Inset: the random 30-dimensional Ising model (red: *J*_ij_ = 1, blue: *J*_ij_ = −1). **c** An experimental annealing curve of a random 1024-dimensional Ising problem. Dashed line: 87.8% approximate solution. **d** The pre-trained single-layer ANN for MNIST dataset. **e**, **f** The accuracy and confusion matrix of the single-layer ANN performed by computer and the SUANPAN, respectively. **g** The pre-trained double-layer ANN for MNIST dataset. **h**, **i** The accuracy and confusion matrix of the double-layer ANN performed by computer and the SUANPAN, respectively

Specifically, for 4-bit precision, the experimental photocurrent for *a* × *b* is shown in Fig. 3g, where *a* remains unchanged and *b* takes 0, ±1, …, ±15. The recorded on-off states of the utilized 8 BEADs are also shown in the bottom inset of Fig. 3g, which is corresponding to the first column of Fig. 3b. Moreover, the fidelities of 4-bit precision with various dimensionalities are shown in Fig. 3h. As the dimensionality increases, the computational fidelity remains above 95%. Due to the high fidelity in executing random signed vector inner products, the SUANPAN architecture can be flexibly utilized to further demonstrate more specific computing tasks. Here, two typical AI tasks are considered, the Ising problem^38^ and ANN, in which the vector multiplication is the core computing operation as shown in Fig. 4a.

An *N*-dimensional Ising problem is defined by a symmetric interaction matrix *J* (*N* × *N* dimensionality with diagonal elements of zero), and the Hamiltonian of Ising problem is defined as follows:

1 $$H={S}^{{\rm{T}}}{JS}$$

Solving Ising problem is to find the specific vector *S* that minimizes the Hamiltonian, which is denoted as the ground state. Here, the simulated annealing (SA) algorithm^39^ is employed, which searches for the ground state through multiple iterations. In each iteration of SA, the variation of Hamiltonian Δ*H* is calculated, which can be transformed into an *N*-dimensional vector inner product and can be readily performed by the SUANPAN (details in Supplementary Note 6). According to the previous reports^40^, a programmable photonic Ising machine with heuristic algorithms has successfully solved the highest dimensionality of 30-dimensional arbitrarily connected Ising problem (detailed discussion and comparison can be found in our previous work^40^). For convenient comparison, a randomly generated 30-dimensional Ising problem is solved experimentally by the SUANPAN. The solving process is repeated with 100 rounds, and the 100 annealing curves are shown in Fig. 4b. It can be seen that 99 curves eventually converged to the ground state (dashed line shown in Fig. 4b), therefore, an accuracy of 99% is achieved by the SUANPAN, which is much higher than the existing 30-dimensional Ising machine based on SA^40^. Further, a randomly generated 1024-dimensional Ising problem is considered. As common practices, an approximate solution with 87.8% of the ground state is set as a criterion for successful solution^41–43^, as dashed line shown in Fig. 4c. Such 1024-dimensional Ising problem is successfully solved by the SUANPAN as the annealing curve fall below the criterion line after ~4000 iterations. The high convergence rate and high dimensionality for solving various Ising problems can validate the programmability, reconfigurability and computational stability of the SUANPAN architecture. Recently, an on-chip Ising machine^44^ is demonstrated with both linear and nonlinear operations in optical domain. Of course, it is beyond the scope of this work. But it is an interesting topic to combine the SUANPAN architecture with nonlinearity, and we are still undergoing it.

For ANN, various physical neural networks (PNNs), including optical neural networks (ONNs), have been applied to accelerate the calculation. In such PNNs, silico training is usually required to avoid errors caused by differences between simulation and practical devices. Unlike that, the SUANPAN can directly map a pre-trained ANN, in which the vector matrix multiplication can be considered as a set of vector inner products and performed in the optical domain, while the nonlinear activation function would be executed by an electronic processor. Therefore, through time-division multiplexing, the SUANPAN can execute ANNs of varied depth and number of nodes in theory. It should be mentioned that it depends on both reconfigurable and programmable abilities of the SUANPAN since the dimensionality cannot be extended with time-division multiplexing for a fixed computing architecture. Here, both single and double layer ANNs are performed as shown in Figs. 4d and g, respectively. MNIST handwritten digit dataset is utilized as dataset, and stochastic gradient descent^45^ (SGD) is utilized as training method. The weights of the single-layer ANN and double-layer ANN are 4-bit precision and 6-bit precision according to simulations, respectively (the details are shown in Supplementary Note 7 and Fig. S13). For single-layer ANN, the confusion matrix of 10,000 pictures in the test dataset calculated by computer and the SUANPAN are shown in Fig. 4e, f, respectively. The approaching classification accuracies are 88.08% and 90.12% for the SUANPAN and computer, respectively. It can be seen that the experimental accuracy is 98% of the simulation accuracy, which is comparable with the previous work^29^. This result indicates that only a little deterioration is introduced by the SUANPAN. While for double-layer, only the first 100 pictures in MNIST test dataset are performed as a preliminary verification. The confusion matrix and accuracy calculated by computer and the SUANPAN are shown in Fig. 4h, i, respectively. It can be noticed that the classification accuracy calculated by the computer is much higher than one-layer, while that calculated by the SUANPAN is lower than one-layer. The reason might be the performance of MoTe_2_ PD array has deteriorated after three months of testing (the details are shown in Supplementary Note 12 and Fig. S16). Anyway, we believe that the above results of ANNs can still validate the feasibility of the SUANPAN architecture.

## Discussion

In this work, we have proposed and demonstrated the photonic SUANPAN architecture to perform the vector inner product operations. As a proof of principle, a SUANPAN with 64 pairs of VCSEL and MoTe_2_ PD is implemented. According to the experimental results, the SUANPAN is capable of achieving high computing fidelities for randomly generated vector inner products, and can be applied on two typical AI tasks of the Ising machine and ANN. There are two main contributions in this work.

Firstly, for the SUANPAN architecture, it breaks through the traditional mindset of obtaining optical matrix transformations through the interaction of light beams. Instead, there is no interaction among those propagating light beams of all BEADs. Therefore, the SUANPAN can be decomposed into BEADs as independent computing units. The scalability, reconfigurability, and programmability of the SUANPAN architecture are only based on the multiplication, recombination and modulation of BEAD without any additional cost. Compared with optical matrix transformations through interaction between light beams, the SUANPAN possesses the following advantages: (1) With massive and industrial multiplication of BEADs, the SUANPAN can theoretically be infinitely scalable. (2) The SUANPAN can be flexibly reconfigured and programmed to perform various specific computing tasks. (3) Only correcting the intensity of light beam is required (details are provided in Supplementary Note 8), and there is no requirement to correct the phase term. (4) Even if one BEAD is broken during fabrication or operation, other BEADs would not be affected, and only the operating dimensionality would be decreased. Although 64 BEADs are operating well in our experimental demonstration, the anti-failure ability of the SUANPAN architecture would be of great significance for future large-scale computing since the yield of massive production cannot be always as 100%.

Secondly, the SUANPAN provides a promising solution for optoelectronic analog-digital hybrid computing. Large-scale DAC and ADC arrays are usually required in optoelectronic computing. However, with Bit Encoding and Analog Detecting paradigm, *M*-bit digital electronic signal is converted to analog within a set of *M* BEADs, while each BEAD only represents 1-bit information. Thus, no DAC is required. At the same time, only one ADC is required to convert the total photocurrent into electronic digital signal. Therefore, the Bit Encoding and Analog Detecting computing paradigm greatly reduces the heavy burden introduced by ADC and DAC. Actually, it is also an important issue for the scalability of the SUANPAN architecture.

Thirdly, we would provide a detailed analysis about the energy consumption. The energy consumption of the SUANPAN would be approximately proportional to the number of bit precision, since each BEAD only encoding 1-bit information, and *M* BEADs are required for *M*-bit quantization. The energy consumption of a single BEAD consists of two parts: the energy consumption of VCSEL and that of the MoTe_2_ PD. At 8-bit precision, the average energy consumption of a single VCSEL is ~2.5 mW, and the average energy consumption of a single PD is ~273.4 nW (the details are shown in Supplementary Note 11). Therefore, the total energy consumption of a BEAD is ~2.5 mW. It can be seen that the main energy consumption comes from the VCSEL. The reason might be due to the beam spreading during propagation, the channel of the PD only received a small part of the light beam, and the rest would be wasted (the radius of the light spot is ~200 μm, while the length of the channel is only 10 μm). If the PD and the light-emitter are integrated into a single chip in the future, the efficiency of the light power can be significantly improved.

Finally, we would provide a detailed analysis about the computing speed for both the current and predictable implementations of the photonic SUANPAN. Since all BEADs operate in parallel, the computing speed would not degrade as the bit precision increases. Considering one BEAD, the computing latency contains encoding time of the emitter (*t*_e_), propagating time of light (*t*_p_), and detecting time of the PD (*t*_d_). The rise time and fall time of the VCSEL are 0.46 ns and 0.54 ns, respectively (as shown in Supplementary Note 9 and Fig. S14). In the current implementation, each VCSEL is operating at 100 MHz. Thus, the encoding time is *t*_e_ = 1 μs for multiplier *a* = 0, 1, …, 100. The distance between VCSEL and PD is about 1.5 m, then the propagating time is about *t*_p_ = 5 ns. The rise time and fall time of the PD are 4.72 μs and 6.59 μs, respectively (as shown in Supplementary Note 10 and Fig. S15). Thus, the detecting time of the PD is *t*_d_ = 6.59 μs, which is the larger one between rise time and fall time. Then, the total computing latency would be *t*_e _+ *t*_p _+ *t*_d_ = 7.59 μs, and the computing speed for one BEAD would be 132 KOPS (operation per second). Since our implementation consists of 64 BEADs, considering 8-bit quantization of multiplier *b*, the computing speed of current SUANPAN would be 1.05 MOPS. Actually, the current implementation is only a prototype of the SUANPAN architecture, and there is still a lot of room to improve the performance. Obviously, both the light-emitter and the PD can be integrated into a single chip with the heterogeneous integration of 2D materials. Then, the imaging system in current setup is not required, and the propagating time would be greatly reduced. For example, if the distance between the emitter and PD is reduced to <1 mm, the propagating time would be *t*_p_ < 3.3 ps. Thus, the computing speed is mainly determined by two factors, the BEAD number and bandwidth. Similar to the development of integrated circuit, the BEAD number could be increased through continuously reducing the size of light-emitter and PD. Also, 3D multilayer stacking integration can be utilized to further expand the dimensionality. Meanwhile, due to the research on various high-speed nano-lasers^46–48^ and nano-detectors^49,50^, the bandwidth of a single BEAD, which is actually determined by the lower one between the emitter and PD, could be readily increased to several tens of gigahertz. As a concrete example, the computing speed could achieve >1 POPS/cm^2^ (for 1-bit quantization) in a single chip for BEAD size <10 μm and BEAD bandwidth >1 GHz. It should be noticed that the aforementioned BEAD size and bandwidth are not very difficult to achieve. For example, the operation bandwidth of 50 GHz is achieved on MoTe_2_ PD^51^. Furthermore, both the VCSEL and PD utilized in each BEAD are polarization-insensitive. If the polarization dimension encoding is also introduced into the SUANPAN architecture through properly manipulating the polarization state of both the emitter and the detector^52^, the computing power could be boosted more. Therefore, we believe that the photonic SUANPAN architecture is very promising as an attractive and practicable linear vector machine in the visible future.

## Materials and methods

### Device fabrication of VCSEL

The 850 nm VCSEL epitaxy structure consists of around 35 pairs of AlGaAs bottom distributed Bragg reflector (DBR) and 25 pairs of AlGaAs top DBR. AlGaAs/InGaAs quantum well is used as the active region. 98% AlGaAs layer is used to form oxide aperture. After epitaxy, the wafer goes through P-metal deposition, inductively couple plasma trench and wet oxidation. The N-metal is deposited on the backside of the N-type substrate to form a common cathode. While the individual emitter in the array is connected to separate anode pads on the edge of the VCSEL array chip by electroplated traces (Fig. S11a).

### Device fabrication of MoTe_2_ PD

The PDs are fabricated on a SiO_2_/Si substrate directly grown with a 10 nm 2H MoTe_2_ layer (detailed fabrication process in refs. ^53,54^). First, the patterns are defined by ultraviolet lithography and transferred to the MoTe_2_/SiO_2_ layer by reactive ion etching (SF_6_ acts as the etching gas). Then, the Cr/Au electrodes (10 nm/50 nm) are fabricated using ultraviolet lithography, deposition and lift-off. The schematic diagram of the preparation process is shown in Fig. S4. To prevent degradation, the PDs are packaged with a 10 nm Al_2_O_3_ layer grown by atomic layer deposition. For subsequent testing, the MoTe_2_ PDs are connected to a self-designed printed circuit board using wire-bonding technology (Fig. S11b).

### Experimental setup

Schematics of the experimental setup are illustrated in Figs.1a and S12. Light from the VCSEL array with a wavelength of 850 nm is focused by a zoom lens onto the MoTe_2_ PD array. The 8 × 8 VCSEL array and the PD array are aligned by the illumination optical path. Electrical and optoelectronic measurements of the fabricated MoTe_2_ PDs are carried out with a semiconductor parameter analyzer (PDA FS380) at room temperature in ambient conditions. The time-resolved photoresponse of the PD is measured by the semiconductor parameter analyzer, and the modulation of the laser (S1FC635PM, 635 nm, Thorlabs) is realized through a function waveform generator (DG4062, RIGOL), which creates square wave pulses.

## Supplementary information

Supplementary information

## Data Availability

The data that support the findings of this study are available within the paper and the Supplementary. Other relevant data are available from the corresponding author on reasonable request.
