# Supplementary material for: SUANPAN: scalable photonic linear vector machine
Source: Light Sci Appl. 2026 Jan 1;15:16. doi: 10.1038/s41377-025-02059-7 (PMC12756334; doi:10.1038/s41377-025-02059-7)
Supplement: Supplementary file 1 — Supplementary information [file 41377_2025_2059_MOESM1_ESM.pdf]

Supplementary information for:

SUANPAN: Scalable Photonic Linear Vector Machine

Ziyue Yang[1,5], Chen Li[1,5], Yuqia Ran[2], Yongzhuo Li[1]✉, Xue Feng[1]✉, Kaiyu Cui[1], Fang Liu[1], Hao Sun[1], Wei Zhang[1], Yu Ye[2], Fei Qiao[1], Jiaxing Wang[3], Cun-Zheng Ning[1,4], Connie J.Chang-Hasnain[3], Yidong Huang[1]✉

[1]Department of Electronic Engineering, Tsinghua University, 100084 Beijing, China.

[2]State Key Laboratory for Mesoscopic Physics and Frontiers Science Center for Nano-Optoelectronics, School of Physics, Peking University, 100871 Beijing, China.

[3]Berxel Photonics Company Ltd., 518071 Shenzhen, China.

[4]College of Integrated Circuits and Optoelectronic Chips, Shenzhen Technology University, 518118 Shenzhen, China.

[5]These authors contributed equally: Ziyue Yang, Chen Li.

✉e-mail: liyongzhuo@tsinghua.edu.cn; x-feng@tsinghua.edu.cn; yidonghuang@tsinghua.edu.cn

Supplementary Note 1:

The encoding of multiplier *a* and the influence of rising edge and falling edge

The multiplier a is encoded on the integral intensity of VCSEL by controlling the duty ratio of driving current, which is done by the digital counter in FPGA according to the system clock, as shown in Fig. S1. Here, the rising edge and falling edge are modeled as linear. Normally, the rising edge and falling edge will affect the integral intensity of pulse modulation. However, if the rising edge and falling edge are symmetrical, the integral intensity will remain unchanged. The experimental rising edge and falling edge of the VCSEL is shown in Fig. S14. In the experiment, the clock cycle is 10 ns, and the encoding time is $t_e = 1$ μs as stated in the Discussion of the main text. While, the rise time and fall time of the VCSEL are 0.46 ns and 0.54 ns, and the rising edge and falling edge are relatively symmetrical. Therefore, the influence of rising edge and falling edge on the modulation accuracy of multiplier a can be ignored (less than 1%).

Supplementary Note 2:

The mechanism to perform signed vector inner product

As shown in Fig. S2, N sets of $2 \times M$ BEADs should be employed to perform signed vector inner product $A \cdot B$. The XOR operation of the sign bits of each multiplier a_i and b_i needs to be calculated in advance, which only takes a short amount of time to complete on traditional microelectronic processor. The on-off states of these $2 \times M$ BEADs in the ith set will be decided by the result of the XOR operation. If the result is zero, which means multiplier a_i and b_i have the same sign, then the M BEADs with positive bias voltage will be used to calculate the multiplication of multiplier a_i and b_i as mentioned above, and the other M BEADs with negative bias voltage will be at off-state, and vice versa.

Supplementary Note 3:

The mechanism to perform complex vector inner product

The complex vector inner product can be split into four real vector inner products as follows:

$$A = Re(A) + j \cdot Im(A)$$

$$B = Re(B) + j \cdot Im(B)$$

$$=> \begin{cases} Re(A \cdot B) = Re(A) \cdot Re(B) - Im(A) \cdot Im(B) \\ Im(A \cdot B) = Re(A) \cdot Im(B) + Im(A) \cdot Re(B) \end{cases}$$

where, Re(X) means the real part of complex vector X, and Im(X) means the imaginary part of the complex vector X. Then the four real vector inner products can be performed by the SUANPAN architecture as shown in Fig. S3.

Supplementary Note 4:

The separation between devices to avoid crosstalk

For Gaussian beams, the amplitude and the intensity decrease in cross-section as follows:

$$E = E(z) \cdot e^{-\frac{r^2}{\omega^2}}$$

$$I = I(z) \cdot e^{-2 \cdot \frac{r^2}{\omega^2}}$$

where, ω is the spot radius, *r* is the distance from the light beam axis. In experiment, there is no fixed requirement for the separation between devices, which depends on the tolerable error for specific computing task. Here, in our experiment, a zoom lens is utilized to focus the light beam of VCSEL as shown in Fig. S12. And the distance between devices (~600 μm) is about three times of the spot radius (~200 μm). Then, the amplitude will decrease to e^{-9}, and the intensity will decrease to e^{-18}. Therefore, the crosstalk between devices can be ignored.

Supplementary Note 5:

The fidelity of random vector inner product

As stated in the main text, 1000 rounds of signed vector inner products for each bit precision (2-bit, 4-bit and 8-bit) are randomly generated and performed by the SUANPAN. The calculation accuracy is evaluated by the fidelity expressed as:

$$\text{Fidelity}(x, y) = \frac{x^{\mathrm{T}} y}{\|x\| \cdot \|y\|}$$

Here, 1000 true values calculated by computer are denoted as a vector x, and the answers calculated by the SUANPAN are denoted as a vector y. The fidelity describes the parallel degree between x and y, while the scaling error can be excluded. The normalized experimental results ($x/\|x\|$ and $y/\|y\|$) for 2-bit, 4-bit and 8-bit are shown in the main text Fig.3d-f, respectively. It can be seen that the answers calculated by the SUANPAN are highly consistent with those calculated by computer, and all of those fidelities for 2-bit, 4-bit and 8-bit precisions are higher than 98%.

Supplementary Note 6:

Ising machine performed on the SUANPAN

Ising problem is a typical combinatorial optimization problem and also known as quadratic unconstrained binary optimization (QUBO) problem. An N-dimensional Ising problem can be defined by an interaction matrix J, which is a symmetric matrix of $N \times N$ dimensionality with diagonal elements of zero. For a given interaction matrix J, the Hamiltonian of Ising problem is defined as follows:

$$H = S^{\mathrm{T}} J S$$

Solving Ising problem is to find the specific vector S that minimizes the Hamiltonian, which is denoted as the ground state. Since the element in S can only take 0 or 1, the dimensionality of the solution space is 2^N for an N-dimensional Ising problem. In order to solve Ising problem efficiently, various heuristic algorithms have been developed and so-called Ising machine have been demonstrated on various computing platforms. Among them, the simulated annealing (SA) algorithm[1] is combined with optical computing platforms to form a photonic Ising machine. Actually, annealing denotes a physical process in condensed matter physics, where the solid is heated up and then cooling to let all particles arrange themselves in the lowest energy ground state. Inspired by that, the SA algorithm is utilized to search for the ground state of Ising problem as follows[1]:

```
begin
    initialize S, T
    repeat
        random i, S_i = 1 - S_i
        calculated ΔH
        if ΔH ≤ 0 then accept
        else if exp(-ΔH/T) > random [0,1) then accept
        annealing T = T × annealing rate
    until stop criterion
end
```

The solution process of SA algorithm consists of initialization and n iterations as shown above. In each iteration, one random element of S is flipped (from 0 to 1 or from 1 to 0) and then the variation of Hamiltonian ΔH is calculated. After that, the vector of S would be accepted or not according to ΔH. Obviously, the Hamiltonian should be calculated in each iteration with $O(N^2)$ computation complexity, which is actually the main computational burden in SA algorithm. While, since J is a symmetric matrix and only one element in S is flipped, the variation of Hamiltonian ΔH can be transformed into an N-dimensional vector inner product as follows:

$$H(S_i = 0) = [S_1 \; S_2 \; \cdots \; S_{i-1} \; 0 \; S_{i+1} \; \cdots \; S_N] \begin{bmatrix} 0 & J_{12} & \cdots & J_{1i} & \cdots & J_{1N} \\ J_{12} & 0 & \cdots & J_{2i} & \cdots & J_{2N} \\ \vdots & \vdots & \ddots & \vdots & \ddots & \vdots \\ J_{1i} & J_{2i} & \cdots & 0 & \cdots & J_{iN} \\ \vdots & \vdots & \ddots & \vdots & \ddots & \vdots \\ J_{1N} & J_{2N} & \cdots & J_{iN} & \cdots & 0 \end{bmatrix} \begin{bmatrix} S_1 \\ S_2 \\ \vdots \\ S_{i-1} \\ 0 \\ S_{i+1} \\ \vdots \\ S_N \end{bmatrix}$$

$$H(S_i=1)=[S_1\ S_2\ \cdots\ S_{i-1}\ 1\ S_{i+1}\ \cdots\ S_N]\begin{bmatrix} 0 & J_{12} & \cdots & J_{1i} & \cdots & J_{1N} \\ J_{12} & 0 & \cdots & J_{2i} & \cdots & J_{2N} \\ \vdots & \vdots & \ddots & \vdots & \ddots & \vdots \\ J_{1i} & J_{2i} & \cdots & 0 & \cdots & J_{iN} \\ \vdots & \vdots & \ddots & \vdots & \ddots & \vdots \\ J_{1N} & J_{2N} & \cdots & J_{iN} & \cdots & 0 \end{bmatrix}\begin{bmatrix} S_1 \\ S_2 \\ \vdots \\ S_{i-1} \\ 1 \\ S_{i+1} \\ \vdots \\ S_N \end{bmatrix}$$

$$\Delta H=\begin{cases} H(S_i=1)-H(S_i=0)=\ 2[S_1\cdots S_{i-1}\ 1\ S_{i+1}\cdots S_N]\cdot\begin{bmatrix} J_{1i} \\ \vdots \\ 0 \\ \vdots \\ J_{iN} \end{bmatrix}, & if\ S_i\text{: } 0\rightarrow 1 \\ H(S_i=0)-H(S_i=1)=-2[S_1\cdots S_{i-1}\ 1\ S_{i+1}\cdots S_N]\cdot\begin{bmatrix} J_{1i} \\ \vdots \\ 0 \\ \vdots \\ J_{iN} \end{bmatrix}, & if\ S_i\text{: } 1\rightarrow 0 \end{cases}$$

Considering S_i is flipped from 0 to 1, then the ΔH is the inner product of the ith column of matrix J and vector S. Otherwise, if S_i is flipped from 1 to 0, then negative sign should be taken. Therefore, such vector inner product can be readily performed on the SUANPAN, while other nonlinear operations are performed on an electronic processor. Since the element in S can only take 0 or 1, the bit precision of b in the SUANPAN is 1-bit quantization. Therefore, the configuration of the SUANPAN is 32 sets with 2 BEADs in each set. For 30-dimensional Ising problem stated in the main text, the SUANPAN can perform the 30-dimensional vector inner product at one time. While for the 1024-dimensional, the required 1024-dimensional vector inner product is decomposed into 32-dimensional one with time-division multiplexing for 32 times. The experimental parameters of initial T, annealing rate and iterations are shown in Table S1.

Supplementary Note 7:

ANN performed on the SUANPAN

The model of the single-layer and double-layer ANN performed in this work are shown in the main text Fig.4d and 4g, respectively. MNIST handwritten digit dataset is utilized as dataset, and stochastic gradient descent[2] (SGD) is utilized as training method. The input data is a vector with 28×28 dimensionality, and then is downsampled to 14×14 dimensionality in the downsampling layer. Next, for single-layer ANN such data goes through one fully connected layer, and the nonlinear activation function is Softmax function. While for double-layer ANN, such data goes through two fully connected layer, and the nonlinear activation functions of hidden layer and output layer are Relu function and Softmax function, respectively. Actually, the fully connected layer can be considered as a vector matrix multiplication of the data vector and the weight matrix, which can be decomposed into vector inner products and performed on the SUANPAN. While, other nonlinear activation functions are calculated on an electronic processor. In order to determine the bit precision of weight, the classification accuracies of single-layer ANN and double-layer ANN with different bit precision of weight are shown in Fig. S13a-b, respectively. Here, linear symmetric quantization is employed. Specifically, if the maximum and minimum value in the weight matrix is x_1 and x_2, respectively, then the quantization step-size for M-bit would be

$$\frac{\max(|x_1|, |x_2|)}{2^M}$$

where $|x|$ means the absolute value of x. Obviously, the high classification accuracy would be achieved by high bit precision, while it also requires high computing power. In this work, 4-bit and 6-bit precision are employed in the single-layer and double-layer ANN, respectively. Therefore, the configuration of the SUANPAN is 8 (5) sets with 8 (12) BEADs in each set for single-layer (double-layer) ANN.

Supplementary Note 8:

Performance and rectification of the SUANPAN

The *L-I-V* curve of each VCSEL is shown in Fig. S5. The output intensity of each VCSEL is shown in Fig. S6, where the output intensity is approximately linearly related to the duty cycle of the driving current. The *I-V* curve of each $MoTe_2$ PD at dark condition is shown in Fig. S7, and the photocurrents of each $MoTe_2$ PD at different light conditions are shown in Fig. S8. It can be seen that the photocurrent is approximately proportional to the duty ratio of the VCSEL driving current, which validates the encoding of a stated in the main text. The photoresponsivity controlled by bias voltage of each $MoTe_2$ PD is shown in Fig. S9. It can be seen that the photoresponsivity is approximately proportional to the bias voltage, which validates the encoding of b stated in the main text. The statistical uniformity of the VCSEL array and $MoTe_2$ PD array are shown in Fig. S10. Even though our fabricated VCSELs and $MoTe_2$ PDs show good uniformity and stability, the output intensity of each VCSEL and the output current of each PD may not be completely consistent under the same conditions due to the fabrication error. Therefore, it is necessary to rectify the entire architecture before performing calculation tasks. Firstly, the output dark current of each PD is adjusted to be consistent by changing the bias voltage on each PD. Secondly, under such bias voltage, the output photocurrent of each PD is adjusted to be consistent by changing the output intensity of each VCSEL. Then, it can be considered that all 64 BEADs are consistent.

It should be mentioned that, though we use DAC in FPGA for testing the *L-I-V* curve and *I-V* curve to rectify, all these rectifications only need once at the beginning, therefore DAC is not required in the subsequent calculation tasks.

Supplementary Note 9:

Time-resolved dynamics of the VCSEL

The rise time is defined as the time of the pulse intensity to increase from 10% to 90%, and the fall time is defined as the time of the pulse intensity to decrease from 90% to 10%. The time-resolved dynamics of the VCSEL is shown in Fig. S14. The rise time and fall time are 0.46 ns and 0.54 ns, respectively.

Supplementary Note 10:

Time-resolved photoresponse of the $MoTe_2$ PD

The rise time is defined as the time of the photocurrent to increase from 10% to 90%, and the fall time is defined as the time of the photocurrent to decrease from 90% to 10%. The time-resolved photoresponse of the $MoTe_2$ PD at 2 V bias voltage is shown in Fig. S15. The rise time and fall time of the PD are 4.72 μs and 6.59 μs, respectively.

Supplementary Note 11:

The energy consumption of the SUANPAN architecture

The energy consumption of the SUANPAN would be approximately proportional to the number of bit precision, since each BEAD only encoding 1-bit information, and M BEADs are required for M-bit quantization. The energy consumption of a single BEAD consists of two parts: the energy consumption of VCSEL and that of the PD.

The energy consumption of VCSEL is directly related to the multiplier a. For example, if $a = 0$, then the energy consumption of VCSEL would be zero. While if $a = 100$, then the energy consumption of VCSEL would be the full energy consumption. Therefore, it is reasonable to calculate an average energy consumption between $a = 0$ and $a = 100$, which would be half of the full energy consumption. According to Fig. S6, the average output power of 64 VCSELs is ~0.375 mW at $a = 100$. According to Fig. S5, an output power of ~0.375 mW corresponds to a driving current of ~5.5 mA and a driving voltage of ~1.836 V. The full energy consumption would be ~10 mW. Therefore, the average energy consumption of each single VCSEL is ~5 mW. Meanwhile, since multiplier b is binary encoded to the on-off states of the BEADs in one set, half of the VCSELs are at off-state on average. Therefore, the average energy consumption of each single VCSEL would be 2.5 mW.

The energy consumption of the $MoTe_2$ PD consists of two parts: static energy consumption and dynamic energy consumption. Firstly, the static energy consumption refers to the energy consumption caused by dark current. Since different bias voltages are set on those PDs during various bit precisions, the static energy consumption is determined by the bit precision. Here, considering 8-bit precision, the dark currents of a set of 8 PDs are about 1000 nA, 500 nA, 250 nA, 125 nA, 62.5 nA, 31.25 nA, 15.625 nA and 7.8125 nA, and the bias voltage of a set of 8 PDs are about 1.6 V, 0.8 V, 0.4 V, 0.2 V, 0.1 V, 0.05 V, 0.025 V and 0.0125 V. Therefore, the average static energy consumption of each single PD is ~267 nW. Secondly, the dynamic energy consumption refers to the energy consumption caused by net photocurrent. Since different net photocurrents under different incident light power, the dynamic energy consumption is determined by multiplier a. Similarly, considering $a = 50$ as an average case, the average net photocurrents of a set of 8 PDs at above bias voltages are about 48 nA, 24 nA, 12 nA, 6 nA, 3 nA, 1.5 nA, 0.75 nA and 0.375 nA. Therefore, the average dynamic energy consumption of each PD is ~12.8 nW. Meanwhile, since multiplier b is binary encoded to the on-off states of the BEADs in one set, half of the VCSELs are at off-state on average. Therefore, the average dynamic energy consumption of each PD would be ~6.4 nW. Therefore, the total energy consumption of each PD would be ~273.4 nW.

In summary, the average energy consumption of VCSEL is ~2.5 mW and the average energy consumption of PD is ~273.4 nW. Therefore, the total energy consumption of a BEAD is about 2.5 mW.

Supplementary Note 12:

The deterioration of $MoTe_2$ PD

Due to the rough packaging process in the laboratory, the $MoTe_2$ material may slowly react with the oxygen and water in the air[3]. After three months of testing, the performance of $MoTe_2$ PD array has degraded. Under the same conditions compared to Fig. S8, the photocurrent of different PDs has deteriorated to different degrees. Some PDs have only a little deterioration as shown in Fig. S16a, while others may have obvious deterioration as shown in Fig. S16b. Thus, such deterioration would result in a lower calculation fidelity. Actually, the calculation fidelity is over 95% for single-layer ANN, while less than 80% for double-layer. It is reasonable to believe that such deterioration induced the poor performance of double-layer ANN compared to single-layer. In the future, through surface protection (for example h-BN) and surface modification, the long-term stability of such 2D materials could be significantly improved[3]. Also, advanced processes such as vacuum or nitrogen filling packaging can be utilized. Specifically, in previous work[4], the $MoTe_2$ device have no deterioration for two years under the protection of h-BN.

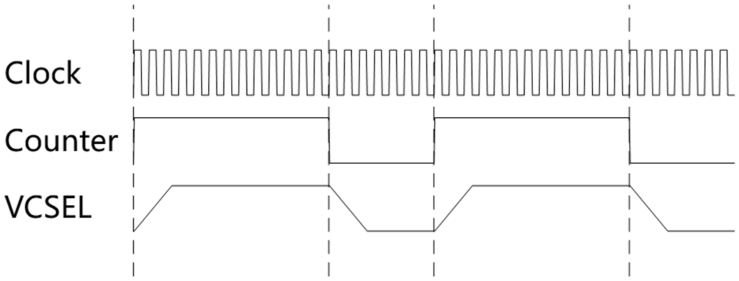

Fig. S1 The encoding of multiplier *a*.

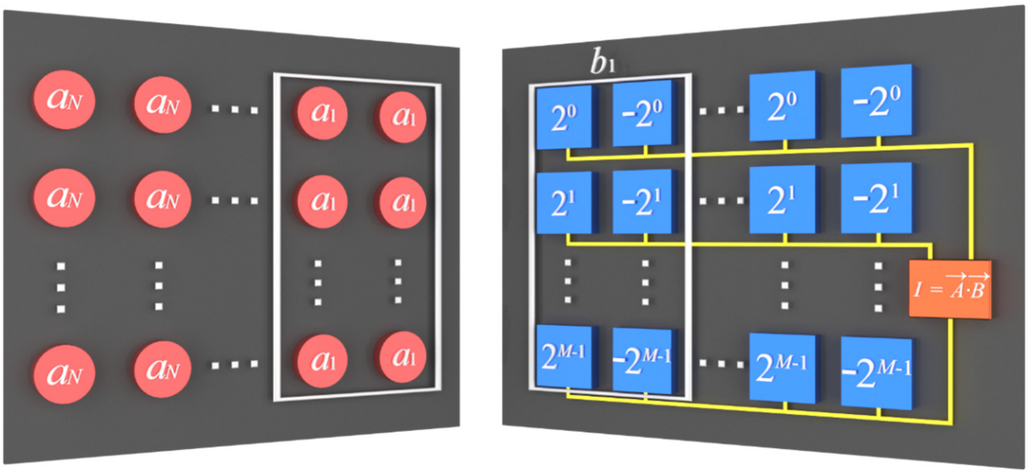

Fig. S2 The mechanism to perform signed vector inner product *A·B* with *N* sets of 2 × *M* BEADs.

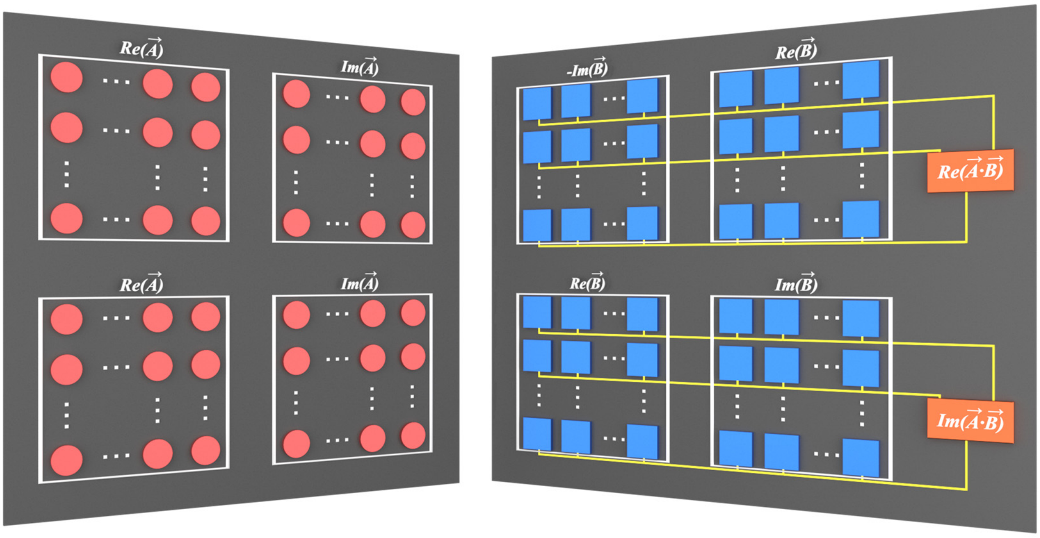

Fig. S3 The mechanism to perform complex vector inner product *A·B*.

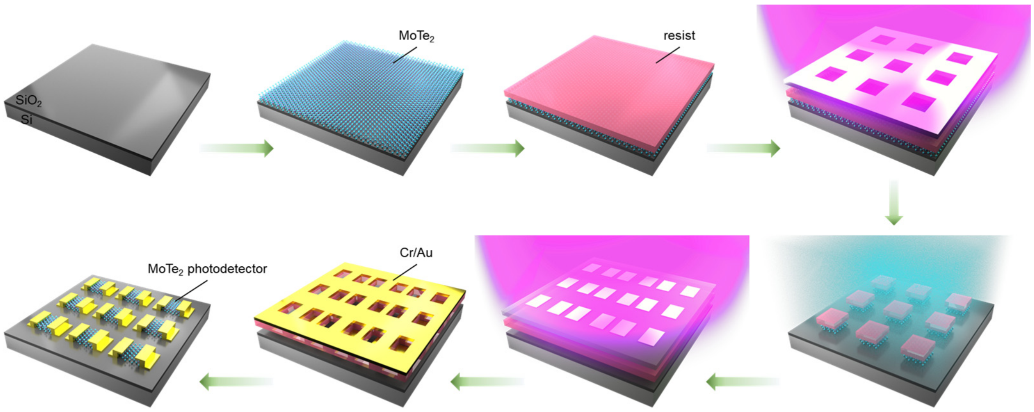

Fig. S4 Schematic fabrication process of $MoTe_2$ PD array.

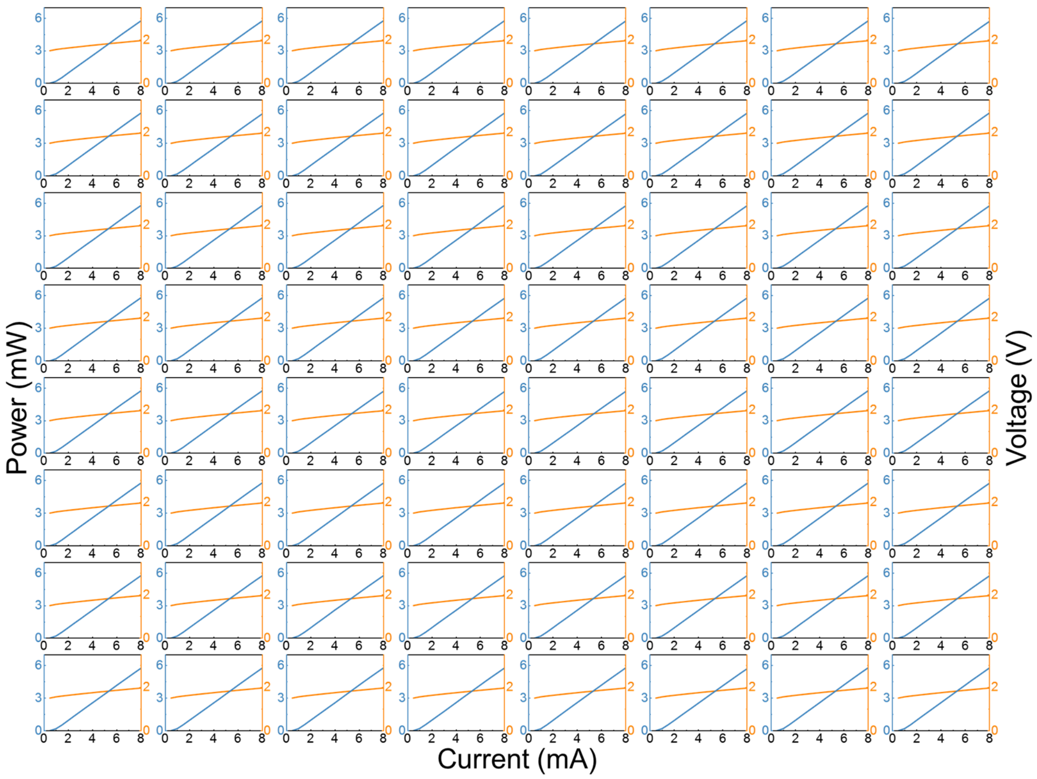

Fig. S5 *L-I-V* curve of each VCSEL.

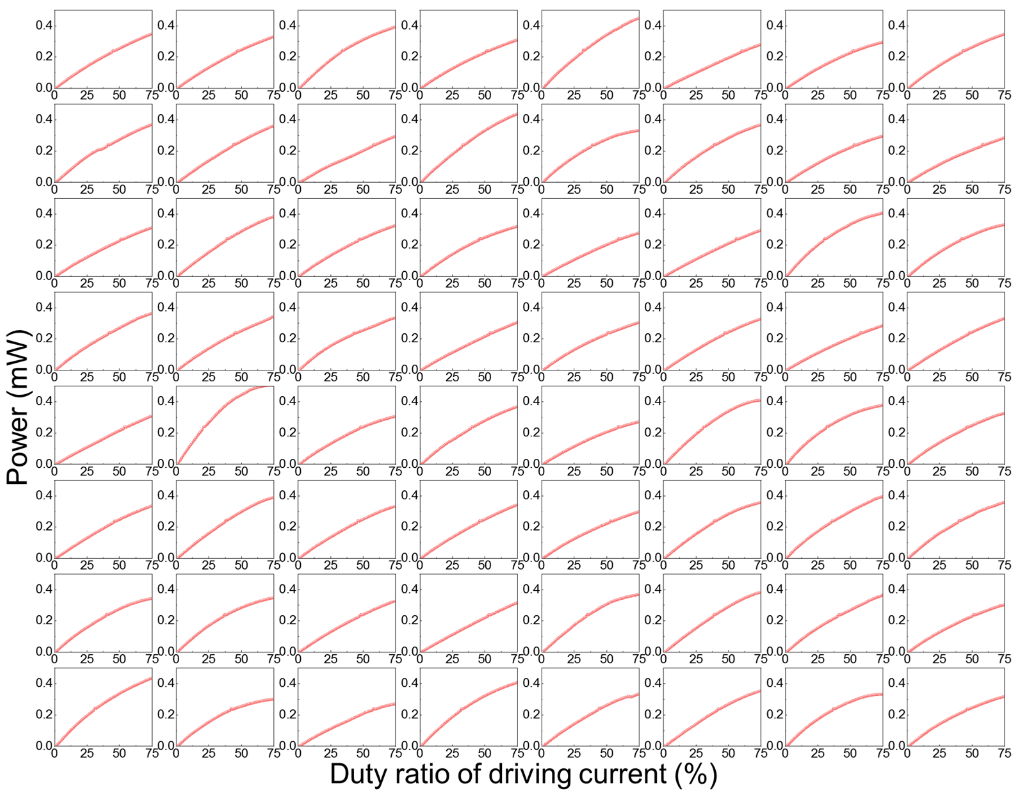

Fig. S6 Output intensity of each VCSEL.

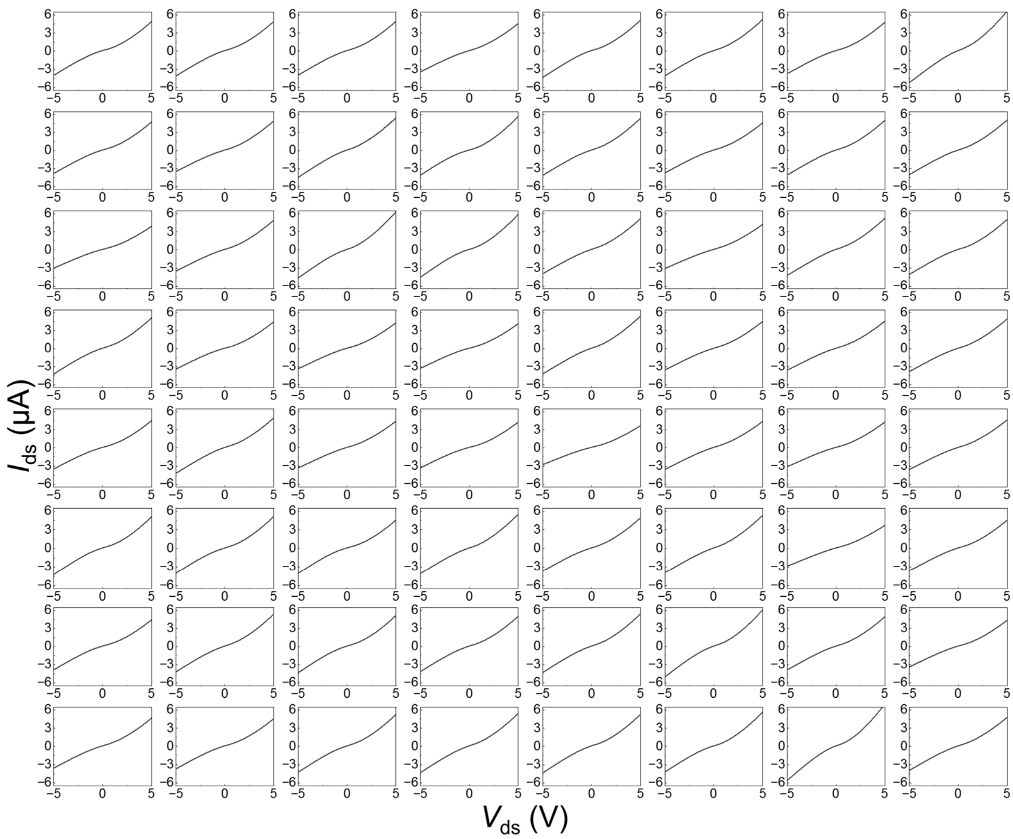

Fig. S7 *I-V* curve of each $MoTe_2$ PD.

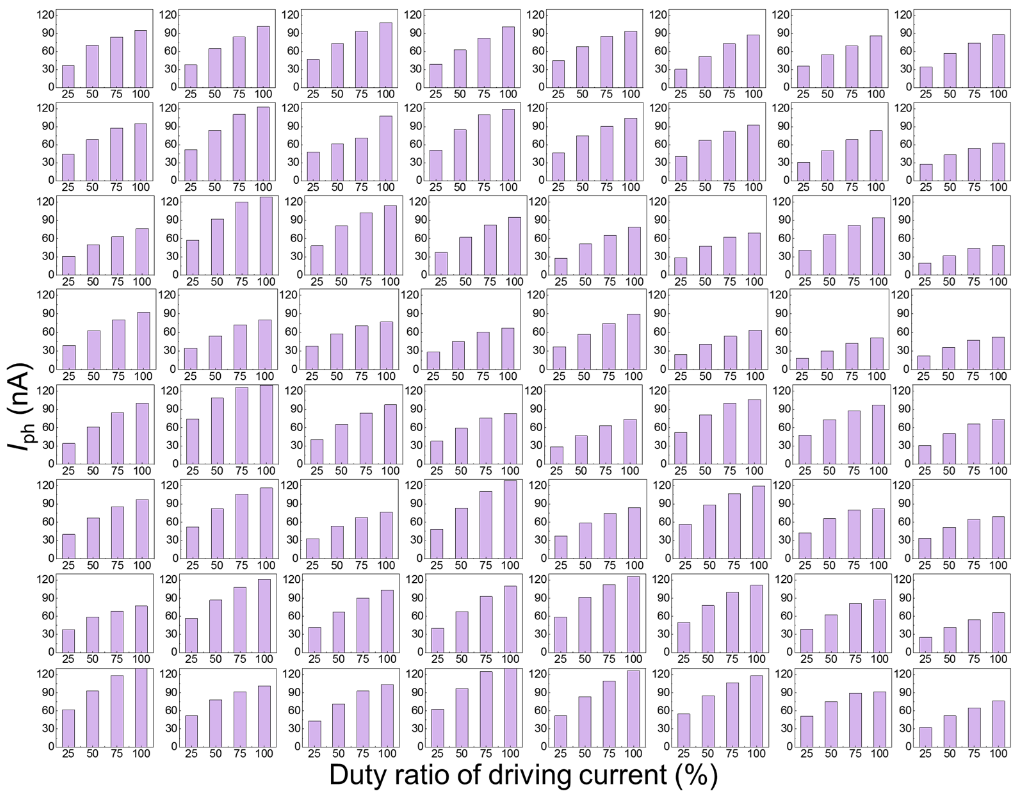

Fig. S8 Photocurrents of each $MoTe_2$ PD at *a* = 25, 50, 75 and 100. For each PD, the bias voltage is 2 V. For each VCSEL, the duty ratio of driving current is 25%, 50%, 75% and 100%.

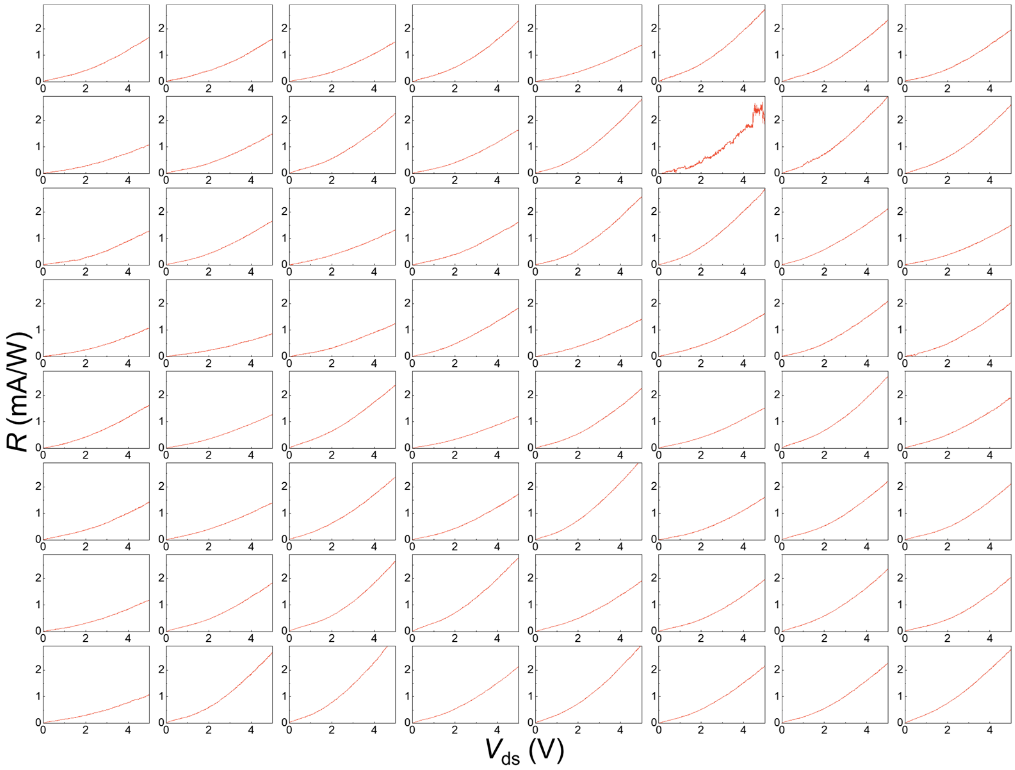

Fig. S9 Photoresponsivity controlled by bias voltage of each $MoTe_2$ PD at *a* = 75. For each VCSEL, the duty ratio of driving current is 75%. For each PD, the bias voltage is controlled from 0 V to 5 V.

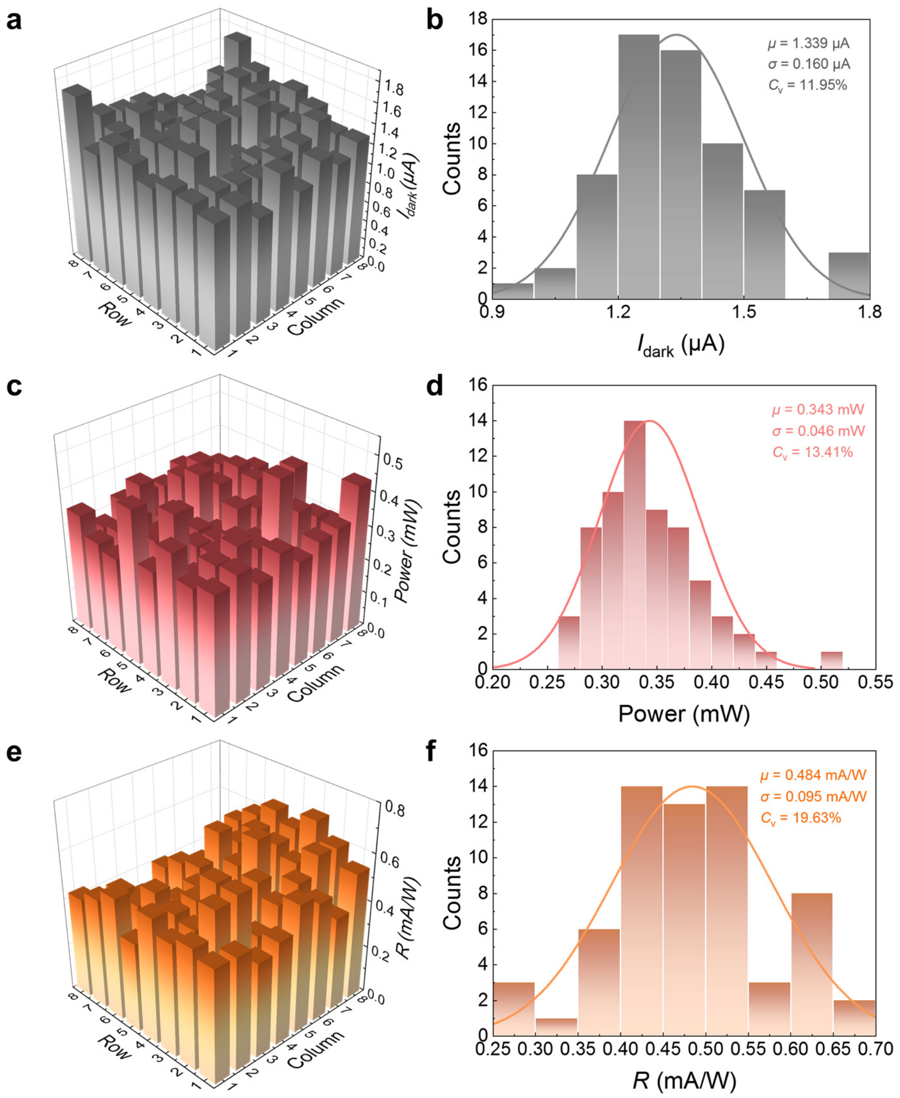

Fig. S10 Statistical Uniformity of the VCSEL array and $MoTe_2$ PD array. a-b The hold-state current mapping and statistical analysis of 8 × 8 $MoTe_2$ PD array without illumination at 2 V bias voltage. **c-d** The hold-state output intensity mapping and statistical analysis of 8 × 8 VCSEL array at 75% duty ratio driving current. **e-f** The hold-state responsivity mapping and statistical analysis of 8 × 8 $MoTe_2$ PD array at 2 V bias voltage.

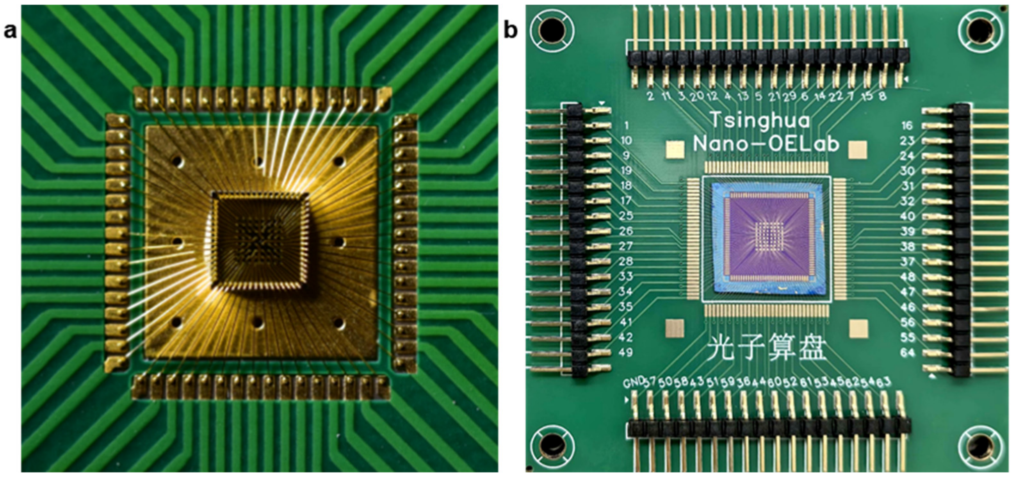

Fig. S11 The VCSEL array (a) and $MoTe_2$ PD array (b) are connected to self-designed printed circuit boards using wire-bonding technology.

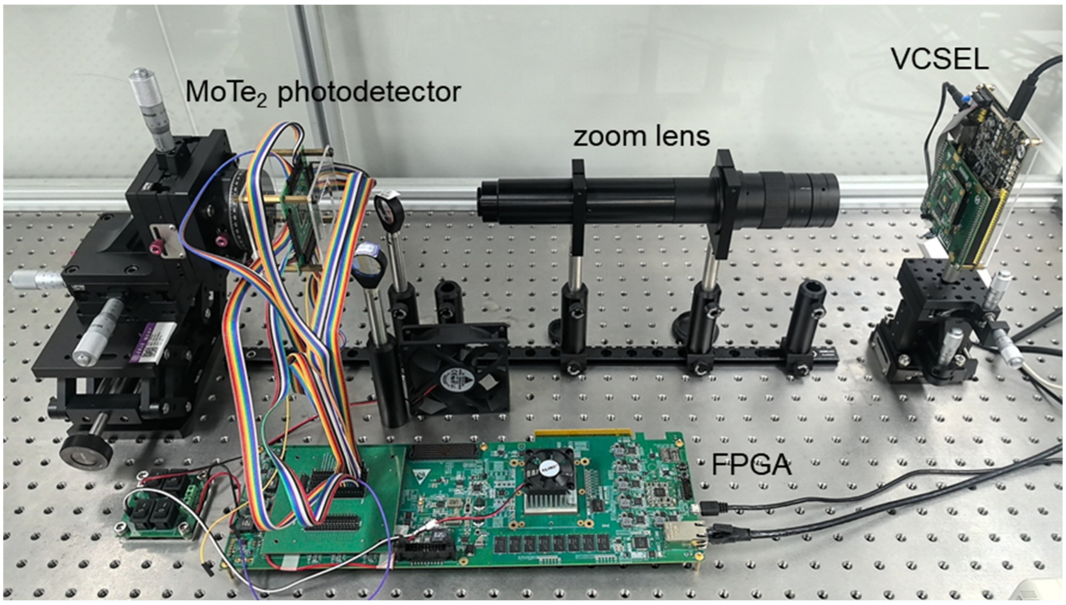

Fig. S12 Optical image of the experimental setup.

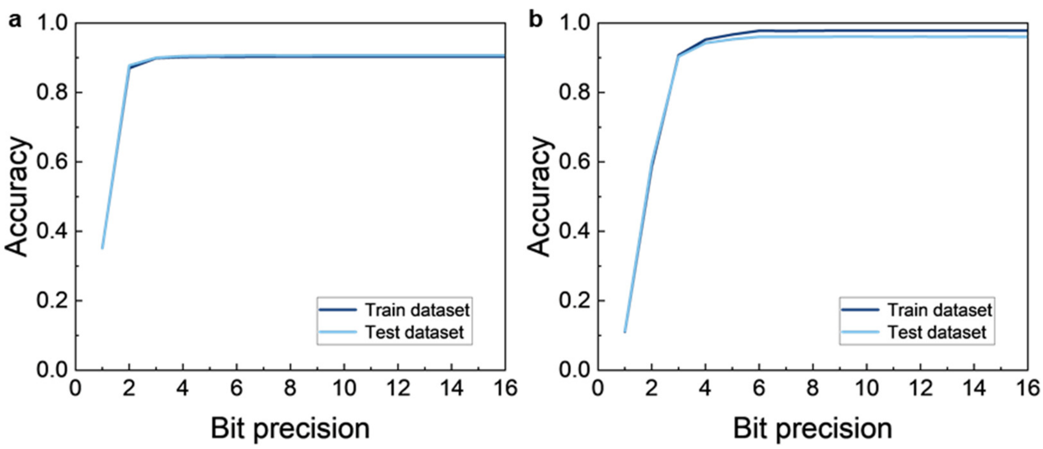

Fig. S13 The quantization of ANN. a The classification accuracies of single-layer ANN with various bit precisions. 4-bit precision is utilized in the experiment of single-layer ANN. **b** The classification accuracies of double-layer ANN with various bit precisions. 6-bit precision is utilized in the experiment of double-layer ANN.

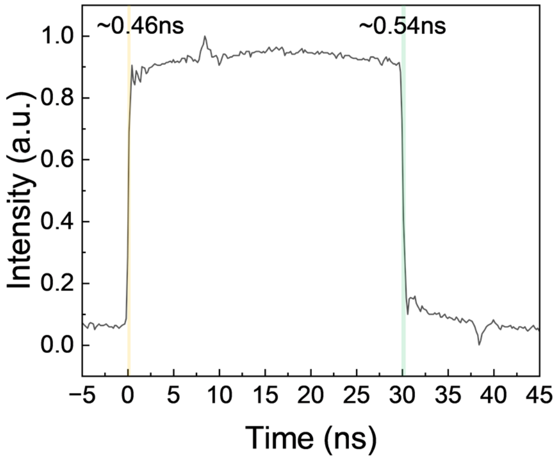

Fig. S14 Time-resolved dynamics of the VCSEL.

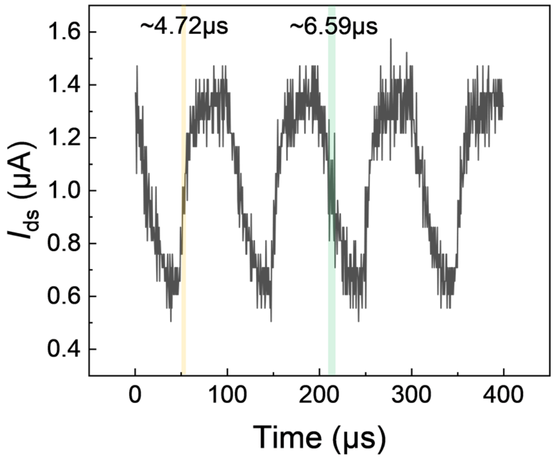

Fig. S15 Time-resolved photoresponse of the $MoTe_2$ PD.

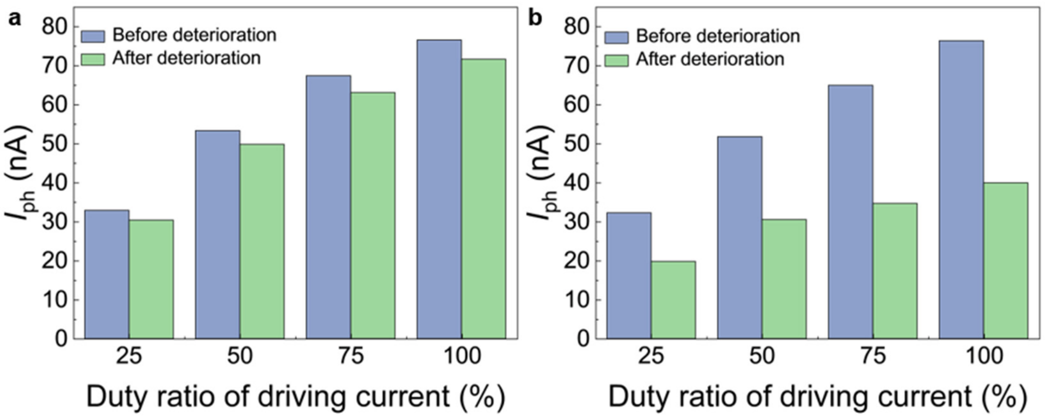

Fig. S16 The deterioration of $MoTe_2$ PD. a An example with weak deterioration. **b** An example with obvious deterioration. (The testing conditions are consistent compared with Fig. S8. For each PD, the bias voltage is 2 V. For each VCSEL, the duty ratio of driving current is 25%, 50%, 75% and 100%.)

Table S1 The parameters of Ising machine.

	Initial temperature	Annealing rate	Iterations
**30-dimensional Ising machine**	10	0.99	500
**1024-dimensional Ising machine**	50	0.995	5000

1 Van Laarhoven, P. J. & Aarts, E. H. *Simulated annealing: theory and application*. (Springer, 1987).

2 Ketkar, N. *Deep learning with Python*. (Springer, 2017).

3 Wang, X. et al. Chemical and structural stability of 2D layered materials. *2D Mater.* **6** (2019).

4 Bie, Y.-Q. et al. A $MoTe_2$-based light-emitting diode and photodetector for silicon photonic integrated circuits. *Nat. Nanotechnol.* **12**, 1124-1129 (2017).
